# Effect of Ticagrelor, a Cytochrome P450 3A4 Inhibitor, on the Pharmacokinetics of Tadalafil in Rats

**DOI:** 10.3390/pharmaceutics11070354

**Published:** 2019-07-20

**Authors:** Young-Guk Na, Jin-Ju Byeon, Hyun Wook Huh, Min-Ki Kim, Young G. Shin, Hong-Ki Lee, Cheong-Weon Cho

**Affiliations:** College of Pharmacy and Institute of Drug Research and Development, Chungnam National University, 99, Daehak-ro, Yuseong-gu, Daejeon 34134, Korea

**Keywords:** tadalafil, ticagrelor, drug-drug interaction, pharmacokinetics, plasma concentration, CYP3A4

## Abstract

Tadalafil is a cytochrome P450 (CYP) 3A4 substrate. Because there are few data on drug-drug interactions, it is advisable to take sufficient consideration when co-administering tadalafil with CYP3A4 inducers or inhibitors. This study was conducted to assess the effect of ticagrelor, a CYP3A4 inhibitor, on the pharmacokinetic properties of tadalafil after oral administration to rats. A total of 20 Sprague–Dawley male rats were randomly divided into the non-pretreated group and ticagrelor-pretreated group, and tadalafil was orally administered to each group after pretreatment with or without ticagrelor. Blood samples were collected at predetermined time points after oral administration of tadalafil. As a result, systemic exposure of tadalafil in the ticagrelor-pretreated group was significantly increased compared to the non-pretreated group (1.61-fold), and the clearance of tadalafil in the ticagrelor-pretreated group was significantly reduced than the non-pretreated group (37%). The prediction of the drug profile through the one-compartment model could explain the differences of pharmacokinetic properties of tadalafil in the non-pretreated and ticagrelor-pretreated groups. This study suggests that ticagrelor reduces a CYP3A-mediated tadalafil metabolism and that tadalafil and a combination regimen with tadalafil and ticagrelor requires dose control and specific pharmacotherapy.

## 1. Introduction

Erectile dysfunction (ED), the most prevalent complaint in males, is the persistent inability to maintain an erection [1]. Various factors, such as age and presence of cardiovascular diseases, influence the incidence of ED [2,3]. Particularly, vascular diseases, such as coronary artery disease (CAD), are related to a high prevalence of ED [4,5,6]. It has been reported that 42% of patients between the ages of 40 and 60 years are affected by this condition [7,8,9]. In addition, a high rate of ED prevalence (~75%) has been investigated in CAD patients [10,11]. The most commonly prescribed oral drugs for ED are the 5-phosphodiesterase (PDE5) inhibitors, and the drugs for CAD are the platelet aggregation inhibitor [12,13]. Therefore, PDE5 may be co-administered to CAD patients already receiving platelet aggregation inhibitors.

Tadalafil (Cialis^®^), the approved PDE5 inhibitor, is used in the treatment of ED and is one of the most frequently prescribed PDE5 inhibitors [14]. In addition, tadalafil exhibits a longer clinical efficacy (up to 36 h) than sildenafil or vardenafil [15,16]. Because of the long duration of action, a once-a-day dose regimen improves the life quality of patients. Tadalafil is classified as a cytochrome P450 (CYP) 3A4 substrate and is mainly metabolized by CYP3A4 to catechol, which is extensively bound to form methyl catechol glucuronide, a major circulating metabolite of tadalafil through methylation [17].

Ticagrelor (Brilinta^®^), the platelet aggregation inhibitor, belongs to P2Y_12_ receptor antagonists and is used for the treatment of CAD [18,19]. It provides the superior and more sustained inhibition of platelet aggregation than clopidogrel, another P2Y_12_ receptor antagonist [20]. Recent studies have reported that ticagrelor acts as an inhibitor of CYP3A4 [21,22]. When ticagrelor was co-administered with atorvastatin, ticagrelor acted as a CYP inhibitor, increasing the maximal plasma concentration of atorvastatin and the area under the plasma concentration-time curve from 0 to infinity by 23% and 36%, respectively [23]. Therefore, drug-drug interaction by co-administration of tadalafil and ticagrelor can inhibit the metabolism of CYP3A4 substrates, such as tadalafil. Despite the possibility of co-administration, the pharmacokinetic interactions between ticagrelor and tadalafil are still uncertain.

Because ticagrelor is an inhibitor of CYP3A4 that accounts for about 15–30% of the total CYP enzyme in humans, the co-administration potentially affects efficacy and safety by altering tadalafil exposure [24]. In healthy male volunteers, the plasma concentrations of tadalafil have been shown to increase with CYP3A4 inhibitors, such as ritonavir [25]. Although tadalafil is well tolerated, patients have experienced side effects, such as headaches, stomachaches, back pain, muscle aches, nasal congestion, redness, limb pain, dizziness, or blurred vision due to the high exposure of tadalafil [26,27]. In other studies, the side effects were increased when doses of tadalafil were higher, indicating that side effects were somewhat related to blood concentration of tadalafil [28,29]. Thus, the combination of tadalafil with ticagrelor also warranted investigation and a drug-drug interaction study should be conducted. However, pharmacokinetic interactions between tadalafil and ticagrelor have not been reported in vivo models.

The objective of our study was to evaluate the effect of ticagrelor on the plasma concentration-time profiles of tadalafil in rats. This study was performed with a parallel design consisting of a non-pretreated group and a ticagrelor-pretreated group. The ticagrelor-pretreated group received oral administration of ticagrelor for seven days of the pretreatment period to inhibit CYP3A. Tadalafil was then orally administered on the seventh day. The non-compartment analysis was performed to analyze the pharmacokinetic profile and the one-compartment model was successfully applied to compare the pharmacokinetics between the two groups. This study assumed that potential drug-drug interactions between ticagrelor and tadalafil could have a clinical impact on the patients.

## 2. Materials and Methods

### 2.1. Chemicals and Reagents

Tadalafil, ticagrelor, and paclitaxel (internal standard (IS)) were kindly obtained from Korea United Pharma Inc. (Seoul, Korea). Dimethyl sulfoxide, formic acid, and polyethylene glycol 400 (PEG 400) were purchased from Sigma–Aldrich (St. Louis, MO, USA). Acetonitrile and methanol were purchased from J.T. Baker (Phillipsburg, NJ, USA). Analytical grade reagents were used throughout this study. Overall, distilled water was used.

### 2.2. LC-MS/MS Analysis of Tadalafil

The concentrations of tadalafil were analyzed by a liquid chromatography tandem-mass spectrometry (LC-MS/MS) system equipped with Agilent 1290 series and Agilent 6495 Triple Quad LC/MS (Agilent Technologies, Santa Clara, CA, USA). A YMC-Triart C18 column (50 × 2.0 mm, 1.9 µm; YMC Inc., Wilmington, NC, USA) was used. The mobile phase was a mixture of 0.2% formic acid in acetonitrile and 0.2% formic acid in distilled water (50:50, *v/v*), and the flow rate was 0.4 mL/min. The temperature of the column and autosampler were set as 30 °C and 4 °C, respectively. The positive ion mode using Agilent jet stream electrospray ionization (AJS-ESI) was applied to record the scan mass spectra. The ion transitions of tadalafil and IS were set as 390.4→268.1 m/z and 876.4→308.1 m/z, respectively, and detected with a multiple reaction monitoring (MRM) mode. The collision energies for tadalafil and IS were 10 V and 30 V, respectively. The cell accelerator voltage was 5 V and the dwell time was set as 200 ms. The source parameters were set as follows: Gas temperature 200 °C, gas flow 14 L/min, nebulizer 20 psi, sheath gas heater 250 °C, sheath gas flow 11 L/min, capillary 3000 V, and nozzle voltage 1500 V.

In this analysis, the most abundant ion transition of tadalafil (390.4→268.1 m/z) was selected to determine the lowest limit of quantification (LLOQ), and the LLOQ of tadalafil was 3 ng/mL. The range of calibration curve of tadalafil was set to 3–6670 ng/mL. The curve was written with a weighted linear regression (1/x) and showed excellent linearity with R^2^ > 0.997. The method has shown accurate and reproducible results within acceptable tolerances (less than 20% coefficient of variation (CV) at LLOQ and less than 15% CV at all other concentrations) [30]. The acquired LC-MS/MS data were processed with Agilent analysis software (Agilent MassHunter Quantitative Software Version B.07.00, Agilent Technologies, Santa Clara, CA, USA).

### 2.3. Animals

All animal experiments were carried out in accordance with the protocol (No. CNU-01167) and the “Guidelines in Use of Animal” approved by Chungnam National University Institutional Animal Care and Use Committee (Daejeon, Korea, 2019). Male Sprague–Dawley rats (aged 7–8 weeks, bodyweight 250–300 g) were purchased from Nara-Biotec (Seoul, Korea). All animals were housed in a dark-light cycle of 12 h at 22 °C and were allowed free access to water and food.

### 2.4. Pharmacokinetic Study

The experiment was performed in a parallel design. A total of 20 rats were randomly divided into two groups. The control group, Group N (n = 10, non-pretreated rats), was orally administered for 7 days only with the vehicle (normal saline/polyethylene glycol 400/dimethyl sulfoxide = 40:20:20). Group T (n = 10, ticagrelor-pretreated rats), an experimental group, received 10 mg/kg of ticagrelor in the vehicle once a day for 7 days orally. Thereafter, on the seventh day, tadalafil (2 mg/kg) was orally administered 30 min after the final administration of the vehicle or ticagrelor. Doses of all samples were calculated according to the weight of the rats and administered using gavage. The dose of ticagrelor and tadalafil given to rats were determined by converting the human doses (90 mg for ticagrelor and 20 mg for tadalafil) to animal doses with body surface area, according to the United States Food and Drug Administration guidelines [31]. Blood (0.3 mL) was collected from the retro-orbital plexus or the jugular vein at predetermined time-points (0, 0.33, 0.67, 1, 1.5, 2, 4, 6, 8, 12, and 24 h) after the oral administration. Samples were centrifuged at 15,000× *g* for 5 min at 4 °C. The plasma was collected and stored at −20 °C until the concentration of tadalafil was analyzed by LC-MS/MS.

### 2.5. Sample Preparation for LC-MS/MS Analysis

Protein precipitation was applied to extract tadalafil from plasma. Briefly, 200 µL of acetonitrile (0.2 *v/v*% formic acid) containing 500 ng/mL of IS was added to 20 µL of the plasma sample. After shaking for 5 min, the mixture was centrifuged at 15,000× *g* for 5 min. The supernatant (150 µL) was transferred and 5 µL of the sample was injected into the LC-MS/MS.

### 2.6. Pharmacokinetic Data Analysis

Pharmacokinetic data were analyzed based on the non-compartment analysis of WinNonlin software 8.1 (Pharsight Corp., Sunnyvale, CA, USA). The maximum plasma concentration of tadalafil (maximum concentration (C_max_)) and the time to reach the maximal plasma concentration (T_max_) of tadalafil were determined from the plasma concentration-time profiles. The area under the plasma concentration vs time curve from 0 to 24 h (AUC_0–24_) was calculated by the linear trapezoidal rule and the area under the plasma concentration vs time curve from 0 h to infinite time (AUC_0–∞_) was estimated by extrapolating time to infinity. The elimination half-life (T_1/2_) and apparent total clearance (CL/F) were determined from the ln 2/elimination rate constant and dose/AUC_0–∞_, respectively [32].

### 2.7. Pharmacokinetic Modeling

Pharmacokinetic modeling of the plasma concentration-time profile of tadalafil was performed using a one-compartment model to investigate the absorption and elimination rates. The parameters of the one-compartment model of tadalafil were estimated based on data from Group N and Group T.

In this model, the shift of the tadalafil amount in the gut compartment and the central compartment is described by the following equations [33]:

$$\frac{dG}{dt}=\;-K_{a}\cdot G$$

$$\frac{dC}{dt}=K_{a}\cdot G-K_{e}\cdot C$$

where *G* and *C* represent the amount of tadalafil in the gut compartment and the central compartment, respectively. *K_a_* indicates the absorption rate constant from the gut compartment to the central compartment. *K_e_* indicates the elimination rate constant from the central compartment to the gut compartment. Each equation was applied to both Group N and Group T data through the classic model of WinNolin software 8.1 (Pharsight Corp., Sunnyvale, CA, USA).

Predicted plasma concentrations of tadalafil (*Con_pred_*) using the one-compartment model were estimated by the following equation [34]:

$$Con_{pred}=\;C/\left(V/F\right)$$

where *V/F* indicates the central volume of distribution of tadalafil.

The fold error versus concentration or time was calculated using the following equation [35]:

$$Fold\;error=\frac{Con_{pred}-Con_{obs}}{Con_{obs}}$$

where *Con_pred_* indicates the predicted concentration of tadalafil from the one-compartment pharmacokinetic modeling and *Con_obs_* indicates the observed concentration of tadalafil from the pharmacokinetic experiment. The fold error within ±2-fold is an acceptable range [35].

### 2.8. Statistical Analysis

Values are represented as mean ± standard deviation (SD). A student’s t-test was applied for statistical significance of differences (*p* < 0.05) and the statistical analysis was performed using GraphPad Prism 8 software (GraphPad Software Inc., La Jolla, CA, USA).

## 3. Results and Discussion

### 3.1. Pharmacokinetic Data

The purpose of our study was to assess the effect of ticagrelor on plasma concentration-time profiles of tadalafil in rats. Drug-drug interactions may occur when CYP inducers or inhibitors are co-administered with the drug metabolized by the CYP enzyme. Several studies have demonstrated that ticagrelor is a CYP3A4 inhibitor and tadalafil is a CYP3A4 substrate [23,25]. Therefore, potential drug-drug interactions between ticagrelor and tadalafil can occur, affecting the pharmacokinetic profile, the efficacy for treatment of erectile dysfunction, and the frequency of side effects.

To identify the potential drug-drug interactions between ticagrelor and tadalafil in rats, ticagrelor was administered once a day for seven days to inhibit CYP3A in rats only for Group T. On the last day of the study, Group N and Group T received 2 mg/kg tadalafil via oral administration to assess the pharmacokinetics effect of ticagrelor on tadalafil. The plasma concentrations of ticagrelor were sufficient to competitively inhibit CYP3A, and the effects of CYP3A inhibition on systemic exposure of tadalafil could be determined [36].

**Figure 1:**
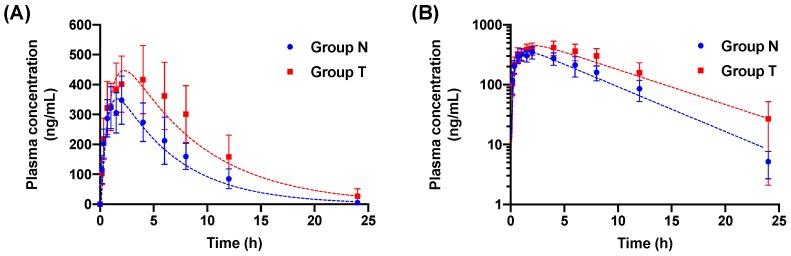
Plasma concentration-time profiles of tadalafil after oral administration to non-pretreated rats (Group N) and ticagrelor-pretreated rats (Group T). Values are represented as mean ± SD (n = 10). Dotted marks and dashed lines indicate the observed plasma concentration and the fitted pharmacokinetic profile from the one-compartment model, respectively. (**A**) Linear scale; (**B**) log scale.

The pharmacokinetic profiles of tadalafil for Group N and Group T are shown in Figure 1. The parameters of the non-compartment analysis are listed in Table 1. The non-compartmental analysis is the model-independent method, which is based on the time course of drug concentrations. Pharmacokinetic parameters from the non-compartmental analysis are mainly used to evaluate drug exposure in oral administration of a drug. The systemic exposure of tadalafil was increased in Group T compared with Group N. Group T showed higher C_max_, AUC_0–24_, and AUC_0-∞_ than the C_max_, AUC_0–24_, and AUC_0-∞_ of Group N. These results showed significant increases in the values of AUC_0–24_ (1.61-fold, *p* < 0.05) and AUC_0–∞_ (1.66-fold, *p* < 0.05). The C_max_ of Group T slightly increased 1.15-fold compared to that of Group N, but there was no significant difference (*p* = 0.3332). The ratio of ACU_0–24_ and AUC_0–∞_ between Group N and Group T exceeded the range of 0.8–1.25, where no pre-specified pharmacological effect was observed [37].

As a result of CYP inhibition by ticagrelor, the plasma concentration of tadalafil decreased more slowly after co-administration of ticagrelor and tadalafil than after tadalafil alone. T_max_, T_1/2_, and CL/F showed statistically significant differences between Groups T and Group N. In the case of T_max_, the absorption of tadalafil in Group T was delayed (T_max_ of 3.22 ± 1.30 h) compared to that in Group N (T_max_ of 1.45 ± 0.50 h) (*p* < 0.005). Thus, the tadalafil plasma concentration remained high until 8 h. This result indicated that the reduced metabolism of tadalafil by CYP3A inhibition caused the absorption of the drug for a longer period of time [38]. In particular, Group T showed an increased T_1/2_ compared to Group N (*p* < 0.005) and the CL/F value of Group T was significantly lower than that of Group N (*p* < 0.005). These results showed that the inhibition of hepatic CYP3A metabolism and the reduction of the first pass effect increased the exposure of tadalafil [39].

**Figure 2:**
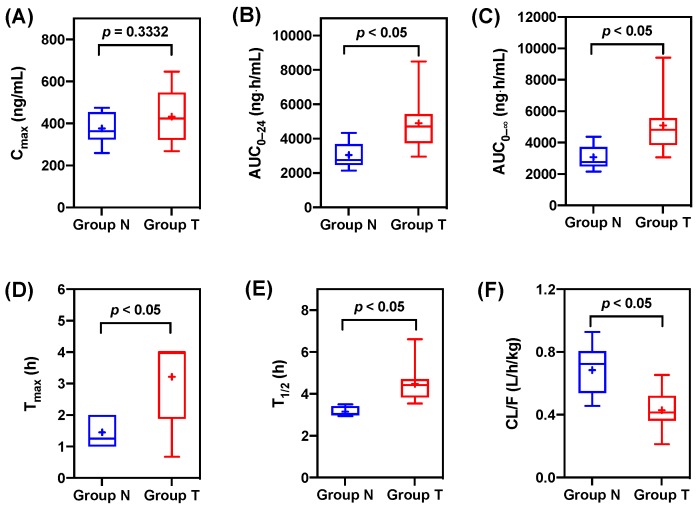
Comparisons of the non-compartment analysis parameters of tadalafil from non-pretreated rats (Group N) and ticagrelor-pretreated rats (Group T). Boxes mean 25th and 75th percentiles of data and whiskers mean 5th and 95th percentiles of data. The median and mean values are displayed as a solid line (–) and a plus mark (+) in boxes, respectively. (**A**) C_max_; (**B**) AUC_0–24_; (**C**) AUC_0–∞_; (**D**) T_max_; (**E**) T_1/2_; (**F**) CL/F. Maximum concentration (C_max_); area under the plasma concentration vs. time curve from 0 to 24 h (AUC_0–24_); area under the plasma concentration vs. time curve from 0 to infinity (AUC_0–∞_);time to reach maximal concentration (T_max_); half-life (T_1/2_); apparent total clearance (CL/F).

In general, the non-compartment analysis parameters of tadalafil showed statistically significant differences between Group N and Group T (Figure 2). In the present study, co-administration with ticagrelor increased tadalafil exposure and affected the AUC_0–24_, AUC_0-∞_, T_max_, and half-life of tadalafil. In particular, the half-life of tadalafil increased from 3.15 h in Group N to 4.47 h in Group T, resulting in the increased AUC_0–24_ (1.61-fold), AUC_0–∞_ (1.66-fold), and T_max_ (2.22-fold). In addition, the result of 37% reduction of tadalafil clearance in Group T compared with Group N supported our findings. These data suggest that ticagrelor inhibits tadalafil metabolism due to drug-drug interaction with tadalafil.

### 3.2. Pharmacokinetic Modeling

The one-compartment model was successfully applied to describe each group in terms of the plasma concentration-time profile after a single oral administration of 2 mg/kg tadalafil. Among the various compartment models, the one-compartment model, which is the simplest and best suited to the observed pharmacokinetic profile of tadalafil, was used to compare the observed pharmacokinetic profile with the fitted pharmacokinetic profile. The one-compartment modeling supports the results of the non-compartment analysis that changed by the co-administration of ticagrelor. The modeling-based comparison allows for greater confidence in the metabolic inhibition effect by comparing the observed pharmacokinetic profile with the fitted pharmacokinetic profile, as well as by matching with the parameters of the non-compartment analysis. Additionally, the model-based approach was used to evaluate a more specific and quantified absorption, elimination, and distribution of tadalafil in vivo.

**Figure 3:**
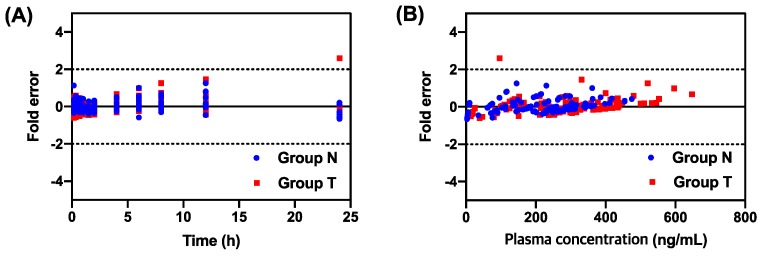
Residual plots written with the pharmacokinetic modeling values of non-pretreated rats (Group N) and ticagrelor-pretreated rats (Group T). (**A**) Fold error vs. time; (**B**) fold error vs. plasma concentration.

As shown in Figure 1, the dashed lines and dotted marks mean the predicted plasma concentration and observed plasma concentration, respectively. The dashed lines showed similar patterns to the dotted marks. Figure 3 indicates the residual plot of the fold error versus time or observed plasma concentration. It shows the acceptable residual values (fold error <2) at most of the analyzed points, which indicates that the one-compartment model is suitable for the explanation of the plasma concentration profile of tadalafil [35,40].

**Figure 4:**
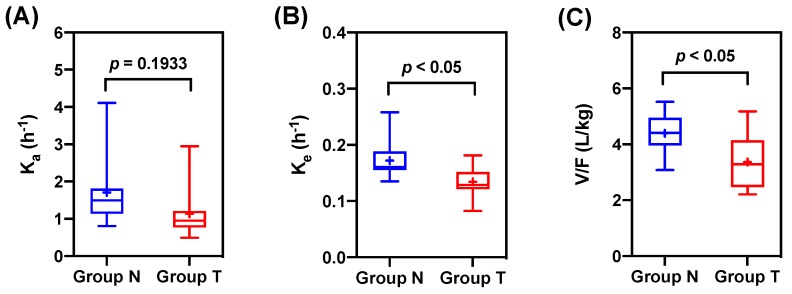
Comparisons of the one-compartment model parameters of tadalafil in non-pretreated rats (Group N) and ticagrelor-pretreated rats (Group T). Boxes mean 25th and 75th percentiles of data and whiskers mean 5th and 95th percentiles of data. The median and mean values are displayed as a solid line (–) and a plus mark (+) in boxes, respectively. (**A**) K_a_; (**B**) K_e_; (**C**) V/F. Absorption rate constant from the gut compartment to the central compartment (K_a_); elimination rate constant from the central compartment to the gut compartment (K_e_); the central volume of distribution (V/F).

Table 2 lists the parameters of the one-compartment model. In the one-compartment model, K_e_ and V/F were decreased significantly in Group T compared to Group N. The K_e_ of Group T (0.13 ± 0.03 h^−1^) were decreased compared with that of Group N (0.17 ± 0.05 h^−1^) (*p* < 0.05). Moreover, the V/F of Group T (3.36 ± 0.95 L/kg) was significantly decreased compared with that of Group N (4.39 ± 0.78 L/kg) (*p* < 0.05). These reductions led to a decrease in the clearance of tadalafil. In addition, the K_a_ of Group T was also decreased, but there was no statistical significance (*p* = 0.1933) (Figure 4). However, this slight reduction was due to the delay in absorption, and it is clear that the absorption increased because of the high C_max_. These results were consistent with those from the non-compartment analysis, indicating that the more absorption and the low clearance caused the increased exposure of tadalafil.

In addition, the results from the modeling indicate that ticagrelor is a weak CYP3A inhibitor. The weak inhibitor is defined as a substance that increases the AUC value of the CYP substrate by 1.25-fold to 2-fold, or that it reduces the clearance of the CYP substrate by 20–50%, and ticagrelor is also classified as this inhibitor [22,41]. Changes in the AUC (1.61-fold) and clearance (37%) of tadalafil by co-administration with ticagrelor were within the range, supporting the above classification. Therefore, we inferred that ticagrelor acts as a weak CYP3A inhibitor, affecting the pharmacokinetic profiles of tadalafil, a CYP3A substrate. Furthermore, co-administration of tadalafil with ticagrelor or other CYP3A inhibitors, such as ketoconazole and diltiazem, would also be expected to increase tadalafil exposure [42].

In a clinical aspect, tadalafil shows robust safety and tolerability [12]. However, the co-administration of tadalafil with ticagrelor to elderly men may cause unexpected side effects. The half-life of tadalafil in normal healthy men is reported to be approximately 17.5 h after administration, whereas that of elderly men is about 21.6 h [43]. This half-life in elderly men is likely to increase further due to co-administration with ticagrelor, which may increase the incidence of side effects, such as headaches and dyspepsia. In addition, patients receiving nitrate medication for treatment of angina are recommended to postpone the nitrate treatment for at least 48 h after administration of tadalafil, but this may need to be longer to prevent serious hypotension [44]. Therefore, most patients receiving these drugs are older, so co-administration of tadalafil should be carefully considered.

## 4. Conclusions

In our study, the plasma concentration-time profile of tadalafil was significantly changed by co-administration with ticagrelor. A ticagrelor-inhibited CYP3A-mediated tadalafil metabolism and the systemic exposure of tadalafil increased by pretreatment with ticagrelor. Co-administration of tadalafil with ticagrelor increased the AUC of tadalafil by approximately 61% and decreased the clearance of tadalafil by 37%. These results suggest that the co-administration of tadalafil and ticagrelor may need dose control and specific drug therapy to avoid side effects from drug-drug interactions. Therefore, CAD patients receiving ticagrelor should be closely monitored when administering tadalafil concomitantly. For further studies, an additional experiment is expected to identify the variation of clinical efficacy of tadalafil by co-administration with ticagrelor, a CYP3A4 inhibitor.

## Figures and Tables

**Table 1:** Non-compartment analysis parameters of tadalafil after oral administration to non-pretreated rats (Group N) and ticagrelor-pretreated rats (Group T). Values are represented as mean ± SD (n = 10).

Parameters	Group N	Group T	Ratio ^a^	*p*-Value
C_max_ (ng/mL)	375.94 ± 72.81	432.71 ± 119.79	1.15	0.3332
AUC_0–24_ (ng⋅h/mL)	3046.88 ± 732.50	4895.74 ± 1592.87	1.61	0.0124
AUC_0–∞_ (ng⋅h/mL)	3070.95 ± 743.01	5095.04 ± 1800.30	1.66	0.0134
T_max_ (h)	1.45 ± 0.50	3.22 ± 1.30	2.22	0.0030
T_1/2_ (h)	3.15 ± 0.23	4.47 ± 0.89	1.42	0.0018
CL/F (L/h/kg)	0.68 ± 0.15	0.43 ± 0.13	0.63	0.0035

^a^ Ratio = $\frac{Value\;of\;Group\;T}{Value\;of\;Group\;N}$; maximum concentration (C_max_); area under the plasma concentration vs. time curve from 0 to 24 h (AUC_0–24_); area under the plasma concentration vs. time curve from 0 to infinity (AUC_0–∞_);time to reach maximal concentration (T_max_); half-life (T_1/2_); apparent total clearance (CL/F).

**Table 2:** The one-compartment model parameters of tadalafil in non-pretreated rats (Group N) and ticagrelor-pretreated rats (Group T). Values are represented as mean ± SD (n = 10).

Parameters	Group N	Group T	Ratio ^a^	*p*-Value
K_a_ (1/h)	1.71 ± 0.91	1.14 ± 0.73	0.67	0.1933
K_e_ (1/h)	0.17 ± 0.03	0.13 ± 0.03	0.77	0.0166
V/F (L/kg)	4.39 ± 0.76	3.36 ± 0.95	0.77	0.0438

^a^ Ratio = $\frac{Value\;of\;Group\;T}{Value\;of\;Group\;N}$; Absorption rate constant from the gut compartment to the central compartment (K_a_); elimination rate constant from the central compartment to the gut compartment (K_e_); the central volume of distribution (V/F).
